# Sequence dependent antitumour efficacy of the vascular disrupting agent ZD6126 in combination with paclitaxel

**DOI:** 10.1038/sj.bjc.6603969

**Published:** 2007-09-11

**Authors:** M Martinelli, K Bonezzi, E Riccardi, E Kuhn, R Frapolli, M Zucchetti, A J Ryan, G Taraboletti, R Giavazzi

**Affiliations:** 1 Laboratory of Biology and Treatment of Metastasis, Department of Oncology, Mario Negri Institute for Pharmacological Research, Bergamo 24125, Italy; 2 Laboratory of Cancer Pharmacology, Department of Oncology, Mario Negri Institute for Pharmacological Research, Milano 20156, Italy; 3 AstraZeneca, Alderley Park, Macclesfield SK10 4TG, UK

**Keywords:** vascular disrupting, microtubule inhibitors, ZD6126, paclitaxel, combination treatments

## Abstract

The clinical success of small-molecule vascular disrupting agents (VDAs) depends on their combination with conventional therapies. Scheduling and sequencing remain key issues in the design of VDA–chemotherapy combination treatments. This study examined the antitumour activity of ZD6126, a microtubule destabilising VDA, in combination with paclitaxel (PTX), a microtubule-stabilising cytotoxic drug, and the influence of schedule and sequence on the efficacy of the combination. Nude mice bearing MDA-MB-435 xenografts received weekly cycles of ZD6126 (200 mg kg^−1^ i.p.) administered at different times before or after PTX (10, 20, and 40 mg kg^−1^ i.v.). ZD6126 given 2 or 24 h after PTX showed no significant benefit, a result that was attributed to a protective effect of PTX against ZD6126-induced vascular damage and tumour necrosis, a hallmark of VDA activity. Paclitaxel counteracting activity was reduced by distancing drug administrations, and ZD6126 given 72 h after PTX potentiated the VDA's antitumour activity. Schedules with ZD6126 given before PTX improved therapeutic activity, which was paralleled by a VDA-induced increase in cell proliferation in the viable tumour tissue. Paclitaxel given 72 h after ZD6126 yielded the best response (50% tumours regressing). A single treatment with ZD6126 followed by weekly administration of PTX was sufficient to achieve a similar response (57% remissions). These findings show that schedule, sequence and timing are crucial in determining the antitumour efficacy of PTX in combination with ZD6126. Induction of tumour necrosis and increased proliferation in the remaining viable tumour tissue could be exploited as readouts to optimise schedules and maximise therapeutic efficacy.

The tumour vasculature is an established target for the therapy of solid tumours. Agents designed to prevent the formation of new tumour vessels (antiangiogenic therapy) or to damage already formed vessels (vascular targeting/disrupting therapy) have been developed and have shown efficacy in preclinical models, and recently, in clinical studies (Taraboletti and Margosio, 2001; Thorpe, 2004; Ferrara and Kerbel, 2005; Tozer *et al*, 2005; Chaplin *et al*, 2006).

The concept of vascular disruption in cancer treatment is based on the differences between vessels of the tumour microenvironment and those of normal tissues: the former are immature, highly permeable, and chaotic with heterogeneous blood flow rates, features that predispose them to the selective action of vascular disrupting agents (VDAs) (reviewed in Thorpe, 2004; Tozer *et al*, 2005). These compounds induce morphologic alterations of the endothelial cells in tumour vessels (Davis *et al*, 2002; Micheletti *et al*, 2003) that trigger a cascade of events ultimately leading to vessel shutdown and tumour necrosis (reviewed in Thorpe, 2004; Tozer *et al*, 2005). Initial events, detected as early as 5–25 min following treatment, include increased permeability to macromolecules, vasoconstriction of tumour supplying arterioles, reduction of blood flow and consequent hypoxia (Horsman and Murata, 2003; Skliarenko *et al*, 2006). Thereafter, platelet activation, coagulation, vessel occlusion, recruitment of inflammatory cells, and direct effects on vascular remodelling may occur, leading to necrosis of the tumour tissue (Blakey *et al*, 2002; Davis *et al*, 2002; Goertz *et al*, 2002; Micheletti *et al*, 2003; Robinson *et al*, 2003). Typically, the final effect of VDAs on tumours is the induction of massive central necrosis (after 24 h), with a rim of viable, proliferating cells remaining at the tumour periphery. These viable tumour cells can rapidly repopulate the tumour, which is then able to resume its growth, unless treatment with the VDA is repeated or the VDA is combined with other types of treatments.

Vascular disrupting compounds include small molecule tubulin-binding agents, flavonoids (DMXAA and FAA), antagonists of junctional proteins, and compounds that target proteins expressed exclusively on the tumour vasculature and are usually used to deliver bioactive molecules (Thorpe, 2004; Neri and Bicknell, 2005; Tozer *et al*, 2005; Chaplin *et al*, 2006). Several tubulin-binding VDAs (CA4P, ZD6126, AVE8062, Oxi-4503, MN-029, ABT-751, and TZT-1027) have been developed and are undergoing preclinical testing and clinical investigation. Among these, ZD6126 is a synthetic water-soluble phosphate prodrug, which is rapidly converted *in vivo* into the tubulin-binding ZD6126 phenol, a microtubule destabilising colchicine analogue. The effects of the compound on endothelial cells *in vitro* and on neo-vessels *in vivo* are well documented (Blakey *et al*, 2002; Davis *et al*, 2002; Micheletti *et al*, 2003), as is its ability to induce tumour necrosis in experimental models (Blakey *et al*, 2002; Siemann and Rojiani, 2002b; McCarty *et al*, 2004; Taraboletti *et al*, 2005; Skliarenko *et al*, 2006).

Preclinical studies have highlighted the potential of using VDAs in combination with conventional therapies, and clinical trials of VDAs in combination regimens are currently underway (reviewed in Chaplin *et al*, 2006). The benefit sought from such combinations is mainly a complementary action between the two agents, with the VDA acting primarily on the tumour vasculature and the chemotherapy or radiotherapy mainly affecting proliferating tumour cells. Moreover, clinical studies have indicated that the toxicity profile of VDAs differs from that of conventional chemotherapy, thus ruling out additive toxicity as a major limitation of the combination. In preclinical studies, ZD6126 has been reported to synergise with radiotherapy (Siemann and Horsman, 2002; Siemann and Rojiani, 2002b; Horsman and Murata, 2003; Wachsberger *et al*, 2005), chemotherapy (Blakey *et al*, 2002; Siemann and Rojiani, 2002a; Goto *et al*, 2004), inhibitors of angiogenesis (Siemann and Shi, 2004), and molecular-targeted agents (Bozec *et al*, 2006).

While confirming the potential benefits of combination treatments, preclinical studies have also underscored the importance of dose, schedule, and sequence to optimise the putative additive effects of combination approaches and to prevent possible negative inhibition (Siemann and Rojiani, 2002b; Wachsberger *et al*, 2005). We have reported previously a potential interfering effect when combining VDAs with chemotherapy agents sharing the same molecular target (tubulin). Taxanes (e.g., paclitaxel (PTX)) are microtubule-stabilising agents, and therefore possess an opposite effect to that of microtubule-destabilising VDAs. In addition, both VDAs and taxanes can act on endothelial cells: by affecting microtubule dynamic instability, taxanes inhibit endothelial cell functions relevant to angiogenesis, thereby acting as antiangiogenic compounds (Belotti *et al*, 1996). We have shown that PTX, given shortly before ZD6126, protected endothelial cells from the activity of the VDA, ultimately preventing its ability to induce vessel occlusion and tumour necrosis in experimental models (Taraboletti *et al*, 2005). We therefore designed the present study to investigate *in vivo* the antitumour activity of ZD6126 in combination with PTX on a human xenograft model, as well as to explore the influence of the drug schedule and sequence on the efficacy of the ZD6126/PTX combination. Tumour responses to the VDA were used as end points to guide the design of the combination with PTX, an approach, which allowed us to optimise conditions for combination regimens.

## MATERIALS AND METHODS

### Tumour cells

The human MDA-MB-435 cancer cell line (Price *et al*, 1990; Sellappan *et al*, 2004) was obtained from the NCI-DCTD Tumour Repository, Frederick, MD, USA. Cells were cultured in DMEM supplemented with 10% fetal bovine serum.

### Animals

Female NCr-nu/nu mice were obtained from the animal production colony of the National Cancer Institute, Frederick Cancer Research and Development Center (Frederick, MD, USA). (Procedures involving animals and their care were conducted in conformity with institutional guidelines, which are in compliance with national (DL no. 116, GU, Suppl. 40, February 18, 1992; *Circolare* no. 8, GU, July, 1994) and international laws and policies (EEC Council Directive 86/609, OJ L 358. 1, December 12, 1987; Standards for the Care and Use of Laboratory Animals, United States National Research Council, Statement of Compliance A5023-01, November 6, 1998.) Mice were used when 8–10 weeks of age (mean body weight=23±2 g). Nude mice were housed in filtered-air laminar flow cabinets and manipulated following aseptic procedures.

### Drug preparation

ZD6126 (MW 437 Da), provided by AstraZeneca, Alderley Park, Macclesfiled, UK, was dissolved in PBS with 0.5% Na_2_CO_3_ and administered i.p. Paclitaxel (PTX, kindly provided by Indena S.p.A., Milan, Italy) was dissolved in 50% Cremophor EL (Sigma, Milan, Italy) and 50% ethanol and further diluted with saline immediately before i.v. administration.

### Antitumour activity

MDA-MB-435 cells (5 × 10^6^) were implanted s.c. in the flanks of nude mice (see explanation in section on ‘Animals’). Tumour growth was monitored two times a week by measuring tumour size with calipers, and estimating tumour weight, in grams, calculated as ((length × width^2^)/2). Treatment started when tumours reached the size of approximately 450 mg, since preliminary results indicated that the activity of VDA is optimal on established tumours (data not shown). Animals were randomised on the basis of tumour weight and subjected to treatment (each group consisted of 6–10 mice). Mice received ZD6126 (i.p.) and PTX (i.v.) at the doses, schedules, and sequence detailed in the Results section. Control mice received the corresponding vehicle. Experiments were concluded when tumours reached a median weight of 2±0.5 g or 4–5 weeks after the last treatment.

Tumour growth was expressed as relative tumour weight RTW=*W*_t_/*W*_o_, where *W*_t_ is the tumour weight at any day of measurement and *W*_o_ was the tumour weight at the start of treatment. Efficacy of the treatment was expressed as %T/C ((median RTW of treated tumours/median RTW of control tumours) × 100). Treatment was considered active when optimal %T/C (the lowest %T/C value for each treatment condition) was lower than 40%. Specific growth delay (SGD) was calculated as ((DT treated – DT control)/DT control), where DT (doubling time) is the time (in days) necessary to double the initial tumour weight (namely to reach a RTW=2). Specific growth delay was considered active when >1. Complete regression was defined as fully regressed tumours, yet undetectable by palpation at the end of the experiment. Statistical differences among groups were evaluated by ANOVA followed by Bonferroni/Dunn *post hoc* test.

### Analysis of tumour necrosis, mitosis, and proliferation

Nude mice were transplanted s.c. with MDA-MB-435 cells as described above. For the evaluation of tumour necrosis, mice bearing tumours of approximately 450 mg were randomised and treated with vehicle or PTX (20 mg kg^−1^, i.v.), followed 2, 24, 72 h, and 1 week later by ZD6126 (200 mg kg^−1^, i.p.) (*n*=5 mice per group). Tumours were then removed 24 h after treatment with ZD6126, cut sagittally along the midline, fixed in formalin and processed for histological analysis. Five-micrometer sections were cut and stained with haematoxylin and eosin (H&E) following standard procedures. Images of the whole sagittal H&E-stained tumour sections were captured using a Nikon Super Coolscan 4000 ED film scanner (Nikon, Tokyo, Japan). Necrosis was analysed by computerised image analysis (Image Pro-Plus 4.5, Media Cybernetics, Silver Spring, MD, USA), exploiting the difference in staining intensity between vital and necrotic tissue (Taraboletti *et al*, 2005). Area of necrosis was expressed as the percentage of total tumour area. Presence of necrosis was also confirmed by histopathological analysis.

For the evaluation of ZD6126-induced cell proliferation, MDA-MB-435 tumours were collected 24 h after administration of ZD6126 (200 mg kg^−1^, i.p.) or vehicle (*n*=5 mice per group), fixed and stained with H&E as above or immunostained for Ki-67. Antibodies against Ki-67 (clone MM1, Ventana Medical Systems, Tucson, AZ, USA) were used according to the manufacturers' specifications. Negative control included omission of the primary antibody and incubation with non-immune serum. Background immunostaining was found to be negligible when non-immune serum was substituted for specific antiserum. Immunostaining was visualised by the avidin–biotin–peroxidase complex method using the Vectastain Elite ABC kit (Vector Laboratories, Burlingame, CA, USA) with 2,2′-diaminobenzidine as a substrate. Haematoxylin was used to counterstain the nuclei of the specimens after immunostaining. Positively stained cells were identified by the presence of a brown nuclear precipitate. Negative cells were identified by the absence of precipitate and only blue counterstain.

For each H&E- and Ki-67-stained section, five random non-overlapping fields at a magnification of × 400 (high-power field (HPF)) in the viable areas of tumours were captured with an Olympus Camedia C-3030 Zoom digital camera (Olympus, Tokyo, Japan). Images were analysed with Image Pro-Plus. Mitotic figures and total cell number were counted in five HPFs/tumours, and the mitotic index was expressed as the number of mitoses per 500 cells. The proliferation index was expressed as the percentage of Ki-67-positive nuclei per total number of cells. Statistical differences among groups were evaluated by ANOVA followed by Bonferroni/Dunn *post hoc* test (comparison between more than two groups) or Mann–Whitney *U*-test (comparison between two groups).

## RESULTS

### Paclitaxel administered before ZD6126 prevents its vascular disrupting activity

We first evaluated the effect of combination regimens with ZD6126 given after PTX. The rationale for this schedule was that the vascular effect of the VDA would cause ‘trapping’ of the already present cytotoxic drug within the tumour, and, at the same time, that the possible VDA-induced impairment of PTX distribution in the tumour would be avoided (Chaplin *et al*, 2006).

**Figure 1:**
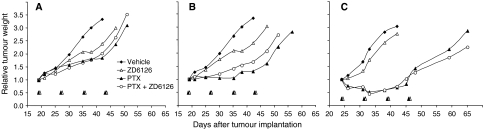
Effect of paclitaxel (PTX) given 2 h before the administration of ZD6126. Mice bearing MDA-MB-435 xenografts (approximately 450 mg) were treated weekly with PTX, ZD6126 or the combination for four courses. Paclitaxel was administered i.v. at the dose of (**A**) 10 mg kg^−1^, (**B**) 20 mg kg^−1^, and (**C**) 40 mg kg^−1^ followed, after 2 h, by ZD6126 (200 mg kg^−1^ i.p.). Mice not receiving drugs were treated with the corresponding vehicle at the same times. *n*=7–8. Black (PTX) and white (ZD6126) arrowheads indicate treatments.

Figure 1 shows the antitumour activity of a combination regimen with ZD6126 given shortly (2 h) after PTX on the growth of established MDA-MB-435 xenografts. Paclitaxel alone, given at 10, 20, and 40 mg kg^−1^, caused a dose-dependent inhibition of tumour growth, with a %T/C of 55.5, 38.9, and 20.2, respectively (Figure 1). ZD6126 alone (200 mg kg^−1^) modestly affected the tumour growth with a %T/C ranging from 68.7 to 76.7. The administration of ZD6126 2 h after PTX treatment showed no significant additional benefit compared with PTX alone. This was evident at all doses of PTX (%T/C=59.0, 49.9, and 20.6, respectively, for the combination with PTX at 10, 20, and 40 mg kg^−1^).

The lack of potentiating effect of this combination schedule is in agreement with the reported interference between the two agents, which have an opposite effect on the same target, that is, microtubules (Taraboletti *et al*, 2005). We have previously shown that PTX, which stabilises microtubules, reversibly protects endothelial cells from the microtubule destabilising activity of ZD6126, thereby preventing microtubule depolymerisation, vessel shutdown, and tumour necrosis induced by the VDA (Taraboletti *et al*, 2005). This protective effect was due to the opposite actions of the two drugs on microtubules (Taraboletti *et al*, 2005). A similar combination schedule of ZD6126 with a tubulin-binding compound having microtubule destabilising properties, namely, vincristine, was more effective in damaging endothelial cells than the single agents (Taraboletti *et al*, 2005) and did not interfere with ZD6126-induced tumour necrosis (not shown), thus confirming that interference occurred specifically with PTX.

**Figure 2:**
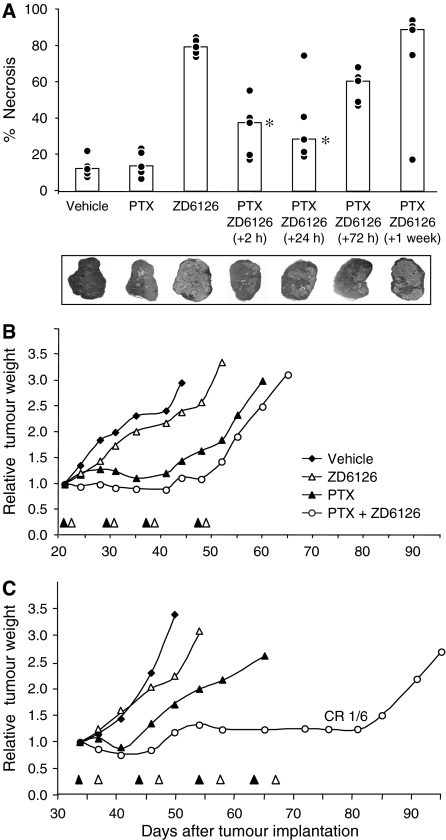
Effect of paclitaxel (PTX) on ZD6126-induced necrosis parallels tumour response. (**A**) MDA-MB-435 cells were xenografted in nude mice. When the tumours reached approximately 450 mg. mice were treated i.v. with PTX (20 mg kg^−1^) given 2, 24, 72 h, and 1 week prior to ZD6126 (200 mg kg^−1^ i.p.). Twenty-four hours after ZD6126 treatment tumours were excised, sections stained with haematoxylin and eosin (H&E) and the percentage of necrotic area calculated as described in Materials and Methods section. Columns indicate the median value. ^*^P=0.01 compared to ZD6126 alone (ANOVA followed by Bonferroni/Dunn *post hoc* test). (**B** and **C**) Mice bearing MDA-MB-435 tumours (approximately 450 mg) were treated with PTX (20 mg kg^−1^ i.v.) 24 h (**B**) or 72 h (**C**) before ZD6126 (200 mg kg^−1^ i.p.). Mice received weekly cycles of treatment for four courses. *n*=6. Black (PTX) and white (ZD6126) arrowheads indicate treatments. CR=complete remission (cured mice, remaining disease free for at least 4 weeks after the end of treatment).

Since prevention of VDA activity by PTX was reversible (Taraboletti *et al*, 2005), we hypothesised that by increasing the time interval between the administrations of the two agents, the interference between the two drugs would be overcome, and eventually the antineoplastic activity of the combination improved. To determine the timing conditions that minimise the protective effect of PTX, we used as readout the analysis of central tumour necrosis caused by ZD6126 (Taraboletti *et al*, 2005). In MDA-MB-435 tumours, a single injection of ZD6126 alone caused massive central necrosis of the tumour mass, evident 24 h after treatment (6.7 times greater than vehicle), whereas PTX alone had no effect (Figure 2A). ZD6126-induced necrosis was prevented by administering PTX (20 mg kg^−1^) 2 or 24 h before the VDA (Figure 2A). However, when the interval between PTX and ZD6126 administration was lengthened, the protective effect of PTX was reduced (72 h) or no longer observed (1 week), and ZD6126 was again able to induce the typical pattern of massive central necrosis surrounded by the rim of viable cells at the tumour periphery (Figure 2A, inset). This finding indicated that drug interference might be overcome by allowing a time interval of at least 72 h between administrations of PTX and the VDA. Indeed, ZD6126 given 24 h after PTX showed no significant benefit compared to administration of the single agents (Figure 2B), whereas ZD6126 given 72 h after PTX resulted in greater antitumour activity than each single agent, generating an increase in doubling time (10.1, 12.0, 20.1, and 55.6 days for vehicle, ZD6126, PTX and the combination, respectively) and SGD (1.0 and 4.5 for PTX and the combination, respectively (Figure 2C). One out of six mice in this group was tumour free at the end of the study.

### ZD6126 administered before paclitaxel potentiates antitumour activity

**Figure 3:**
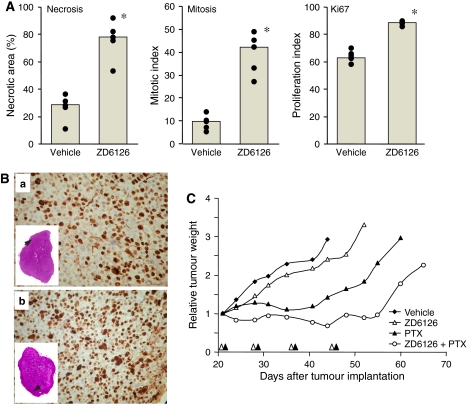
Antitumour effect of paclitaxel (PTX) given after ZD6126. (**A**) Mice bearing MDA-MB-435 tumour xenografts were treated with ZD6126 (200 mg kg^−1^ i.p.) or vehicle as described in the legend to Figure 2. Tumours (*n*=5) were removed 24 h later, processed and analysed as described in Materials and Methods section. Necrosis, mitotic index, and proliferation index were evaluated. Symbols indicate values for each tumour (mean of values from five HPFs/tumours), columns are the median. ^*^P=0.004 compared to vehicle (Mann–Whitney *U*-test). (**B**) Representative images of Ki-67 stained sections of tumours treated with vehicle (a) or ZD6126 (b). Insets show the whole sagittal area of the tumour (original magnification × 10), arrows indicate the viable area where mitosis and proliferation were analysed. (**C**) Mice bearing MDA-MB-435 tumours (approximately 450 mg) were treated with PTX (20 mg kg^−1^ i.v.) 24 h after ZD6126 (200 mg kg^−1^ i.p.). Mice received weekly cycles of treatments for four courses. *n*=6–10. Black (PTX) and white (ZD6126) arrowheads indicate treatments. See for comparison the opposite schedule (namely, PTX given 24 h before ZD6126) in Figure 2B.

In a second set of experiments, we investigated the inverse combination schedule, namely giving the ZD6126 before PTX. The rationale underlying this schedule was that VDA-treated tumours rapidly regrow from the rim of residual viable cells at the tumour periphery that shows prominent proliferative activity (Thorpe, 2004; Tozer *et al*, 2005). Highly proliferating cells are ideal targets for cytotoxic drugs, a premise that underpins the hypothesis that pre-administration of the VDA might generate favourable conditions for the activity of chemotherapy. ZD6126-induced increase in proliferative activity at the tumour periphery was measured as the expression of Ki-67, a marker of cell proliferation, and the mitotic index in the periphery of MDA-MB-435 tumours after treatment. As shown in Figures 3A and B, concomitant with the induction of massive necrosis, ZD6126 caused a significant increase in mitosis and proliferation index 24 h after administration. These findings provided the rationale for administering the cytotoxic drug 24 h after the VDA. Indeed, the combination of ZD6126 with PTX given 24 h later resulted in a significant increase in doubling time compared to each single agent (10.3, 13.8, 32.1, and 41.2 days for vehicle, ZD6126, PTX, and ZD6126+PTX, respectively, *P*=0.02) and efficacy (%T/C was 39.5 and 23.5 for PTX and ZD6126+PTX, respectively). Specific growth delay was 2.1 and 3.0 for PTX and the combination, respectively (Figure 3C).

**Figure 4:**
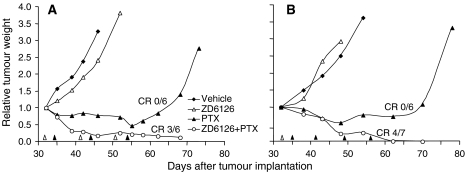
Effect of single versus multiple doses of ZD6126 in combination with paclitaxel (PTX). Mice bearing MDA-MB-435 tumours (approximately 450 mg) were treated (**A**) with ZD6126 (200 mg kg^−1^, i.p.) followed 72 h later by PTX (40 mg kg^−1^, i.v.), for four weekly cycles or (**B**) with a single injection of ZD6126 (200 mg kg^−1^, i.p.) followed by four weekly treatments with PTX (40 mg kg^−1^ i.v.) *n*=6–7. Treatments are indicated by black (PTX) and white (ZD6126) triangles. CR=complete remission (cured mice, remaining disease free for at least 4 weeks after the end of treatment).

The potential drawback of this schedule is that pretreatment with the VDA impairs the functional properties of the tumour vasculature, hence possibly affecting the distribution of PTX within the tumour. Since the activity of this class of molecules, including ZD6126, is rapid and reversible (Micheletti *et al*, 2003), scheduling a sufficient time interval after ZD6126 administration might reasonably avert this possible effect. Figure 4A shows that administration of PTX 72 h after ZD6126 improved the activity of the combination (%T/C=5.4) compared to PTX alone (%T/C=16.4) or ZD6126 (%T/C=72.9). In this experiment, despite the high dose of PTX (40 mg kg^−1^, close to the maximal tolerated dose), tumours in all mice treated with PTX alone eventually regrew, and only in the group receiving the ZD6126/PTX combination were complete remissions observed (three out of six).

Given their mechanism of action, VDAs – unlike most angiogenesis inhibitors – are designed for acute treatments. In this regard, it is worth noting that a single administration of ZD6126 followed by repeated, weekly administrations of PTX was sufficient to produce a degree of antineoplastic activity similar to what was achieved when it was given intermittently in a weekly schedule with PTX (Figure 4B). As expected, ZD6126 did not exert any antineoplastic activity by itself under these conditions, but it was still able to potentiate the activity of PTX, leading to four out seven complete remissions (Figure 4B).

In all the above experiments, animals showed no evident signs of toxicity with the combination treatments.

## DISCUSSION

Vascular disrupting agents given as single agents have shown poor antineoplastic activity, whereas their combination with chemotherapy has yielded promising responses in preclinical models (Horsman and Siemann, 2006). Currently, VDAs are being evaluated primarily in phase II clinical trials in combination with conventional cytotoxic therapies; combinations with other forms of therapy are also under investigation (Chaplin *et al*, 2006). Timing and sequencing of VDA and chemotherapy administration are important issues in such treatments. Here, we show that the antineoplastic efficacy of the combination of a VDA (ZD6126) with a chemotherapeutic (paclitaxel) depends on the sequence and schedule of drug administration. We likewise advance some possible rationales for optimising the combination.

Our previous findings indicated that sequence becomes a crucial issue when combining VDAs with other tubulin-binding agents. Paclitaxel protects endothelial cells from the microtubule destabilising effects of ZD6126 by exerting an opposite biological effect through microtubule stabilisation, thereby inhibiting the ability of ZD6126 to induce vessel shutdown and tumour necrosis (Taraboletti *et al*, 2005). The antagonism between the two agents implies that PTX administered before ZD6126 would prevent the activity of the VDA, thus nullifying the potential of the combination. Indeed, the present study confirmed previous *in vitro* data showing that, regardless of the dose of the chemotherapeutic, ZD6126 given shortly after PTX did not improve the antitumour activity of chemotherapy alone. The finding that tumours pretreated with PTX were made unsusceptible to VDA-induced necrosis confirmed that the lack of activity of the combination was likely due to an antagonistic effect of PTX on the vascular disrupting activity of ZD6126. In keeping with our previous finding that the protective effect of PTX was reversible (Taraboletti *et al*, 2005), we found that increasing the time interval between administration of PTX and VDA resulted in increased antineoplastic activity of the combination. This kinetics of the antitumour activity was closely paralleled by the restored sensitivity of tumours to the vascular disrupting activity of ZD6126 (as shown by induction of necrosis). The association between the response to necrosis and the enhanced antineoplastic activity of the combination indicates that induction of necrosis is a reliable marker of VDA activity, useful when designing combination therapies with potentially antagonistic agents. Imaging techniques, such as MRI or PET, might be suitable tools to assess the optimal time for VDA delivery after chemotherapy.

In addition to being a cytotoxic tumour drug, PTX strongly affects endothelial cells (Belotti *et al*, 1996), which are the primary cellular target of ZD6126 in the tumour microenvironment. It is plausible that optimisation of sequence and timing varies depending on the mechanism of action and cellular targets of the drugs in the combinations. We have previously shown that combinations of VDAs with agents that have the same molecular activity (i.e., tubulin depolymerisation) have a synergic effect on endothelial cell morphology and tumour necrosis (Taraboletti *et al*, 2005). In the same study, we found that, *in vitro*, other chemotherapeutics, such as cisplatin, do not interfere with the activity of the VDA on vascular cells (Taraboletti *et al*, 2005). Nonetheless, sequence and interval between cisplatin and VDA are relevant *in vivo*, where pharmacokinetic interactions between agents may occur (Siemann *et al*, 2002; Siemann and Rojiani, 2002a).

Intuitively, it would seem that VDAs should not be given before chemotherapy, as decreased blood flow and vessel shutdown would decrease the distribution and uptake of chemotherapeutics (Chaplin *et al*, 2006). Nonetheless, our findings indicate that a superior therapeutic efficacy is obtained when ZD6126 is given before PTX (compare Figures 2B and 3C). This might indicate that events elicited by the VDA are relevant to the activity of the chemotherapy given afterward.

It has been proposed that in a combination therapy, VDAs would cause necrosis of the inner part of the tumour, whereas chemotherapy or radiotherapy would target the actively proliferating cells in the peripheral viable rim. We found that ZD6126 can induce changes in the tumour periphery, as shown by the significant increase in cell proliferation associated to the viable tumour tissue (evaluated as the number of mitoses and Ki-67-positive cells) 24 h after its administration. Accordingly, the administration of PTX 24 h after ZD6126 was associated with an increased antineoplastic activity of the combination, suggesting that the mechanism of the favourable effect of pretreatment with ZD6126 might be the increment in the number of proliferating, PTX-responsive cells in the tumour periphery. Moreover, a preliminary pharmacokinetic analysis showed that tumours pretreated with ZD6126 presented an increased ratio between PTX at the tumour periphery and in the inner part of the tumour, suggesting that in VDA-treated tumours the chemotherapeutics concentrate where the tumour is more viable.

The actual target cell in the tumour periphery is still unidentified. Besides tumour cells, endothelial cells might also be targeted by the combination, as shown by the increase in endothelial cell apoptosis induced by the combination of ZD6126 with cisplatin (Goto *et al*, 2004). A recent study (Shaked *et al*, 2006) reported that the VDA OXi-4503 caused a rapid (4 h) mobilisation of circulating endothelial progenitor cells (CEP), which home into the viable rim surrounding the necrotic area, and, 3 days later, are found to be associated with the tumour vasculature. The recruitment of CEP by VDA was prevented by antiangiogenic agents, providing a further rationale for combining VDA and antiangiogenic compounds (Shaked *et al*, 2006). ZD6126, too, mobilises circulating endothelial cells, including CEP (Beaudry *et al*, 2005; Beerepoot *et al*, 2006), and benefits from the combination with antiangiogenic therapies (Siemann and Shi, 2004). However, PTX itself has antiangiogenic properties (Belotti *et al*, 1996), and hence has the potentiality to target CEP (Shaked *et al*, 2005). Taken together, these considerations raise the intriguing possibility that the antineoplastic activity of the ZD6126/PTX combination is the result of the cytotoxic agent targeting CEP recruitment to the tumour periphery following VDA treatment. This hypothesis could also provide a further explanation of our finding that the most favourable antineoplastic effects were obtained with PTX given after ZD6126. In our study, PTX was administered at conventional doses/schedule. It would be interesting to investigate whether low-dose metronomic administration of PTX, reported to act on CEP, would improve the antineoplastic activity of the combination with the VDA.

Our observation that an enhanced combination schedule was obtained by increasing the interval between administration of ZD6126 and of the chemotherapeutic (i.e., 72 h) might indicate that at these conditions a trade-off is achieved between the ‘positive’ effects of VDA on cell proliferation and/or CEP recruitment at the tumour periphery and the ‘negative’ effects of ZD6126 on the distribution of PTX within the tumour. The final outcome of combinations of cytotoxic agents with VDA is therefore the result of each drug's effect on tumour cells and on host tissue compartments (such as tumour blood vessels), and depends on their pharmacological interactions. All these factors account for and should be considered when attempting to optimise schedules and sequence.

Clinical and preclinical studies with ZD6126, as well as with other VDAs, indicate that these compounds are active at well-tolerated doses, well below the MTD. The toxicity profile differs from that observed with cytotoxic chemotherapeutics, indicating that additive toxicity should not be an issue of concern in combination regimens (Horner *et al*, 2004; Beerepoot *et al*, 2006). However, microtubule destabilising VDAs appear to have some characteristic side effects, particularly cardiovascular toxicity (Beerepoot *et al*, 2006; van Heeckeren *et al*, 2006). In addition, VDAs are designed for acute rather than chronic treatments, since their effects in terms of tumour necrosis are apparent within 24 h of treatment. Our finding that significant antineoplastic activity could be achieved with a single VDA treatment followed by repeated administrations of PTX indicates that one treatment is enough to compromise tumour growth, as long as chemotherapy or other forms of treatment contribute to block tumour progression. The effectiveness of this combination schedule has important clinical implications, since some of the possible toxicities of the combined treatment could be limited.

In conclusion, our study indicates that sequencing is crucial in determining the antitumour efficacy of chemotherapy with VDA, and that scheduling of the interval between treatments might be relevant in determining the magnitude of the response. In the case of combinations of tubulin-binding compounds, such as ZD6126 and PTX, VDA-induced necrosis and proliferation index can been used as biological readouts to optimise scheduling of treatments. The best response was obtained by giving the VDA before the tubulin-binding cytotoxic agent and by allowing a significant time interval between the two agents.
